# Crystal structure of pathogenic *Staphylococcus aureus* lipase complex with the anti-obesity drug orlistat

**DOI:** 10.1038/s41598-020-62427-8

**Published:** 2020-03-25

**Authors:** Kengo Kitadokoro, Mutsumi Tanaka, Takaaki Hikima, Yukiko Okuno, Masaki Yamamoto, Shigeki Kamitani

**Affiliations:** 1 Faculty of Molecular Chemistry and Engineering, Graduate School of Science and Technology, Kyoto Institute of Technology, Hashigami-cho, Matsugasaki, Sakyo-ku, Kyoto, 606-8585 Japan; 2 SR Life Science Instrumentation Team, Life Science Research Infrastructure Group, Advanced Photon Technology Division, RIKEN SPring-8 Center, 1-1-1, Koto, Sayo-cho, Sayo-gun, Hyogo, 679-6148 Japan; 3 Medical Research Support Center, Graduate School of Medicine, Kyoto University, Yoshidakonoe, Sakyo-ku, Kyoto, 606-8501 Japan; 4 Graduate School of Comprehensive Rehabilitation, College of Health and Human Sciences, Osaka Prefecture University, 3-7-30 Habikino, Habikino, 583-8555 Osaka, Japan

**Keywords:** X-ray crystallography, Biochemistry, Structural biology, Biophysics, Molecular biophysics, Molecular conformation

## Abstract

*Staphylococcus aureus* lipase (SAL), a triacylglycerol esterase, is an important virulence factor and may be a therapeutic target for infectious diseases. Herein, we determined the 3D structure of native SAL, the mutated S116A inactive form, and the inhibitor complex using the anti-obesity drug orlistat to aid in drug development. The determined crystal structures showed a typical α/β hydrolase motif with a dimeric form. Fatty acids bound near the active site in native SAL and inactive S116A mutant structures. We found that orlistat potently inhibits SAL activity, and it covalently bound to the catalytic Ser116 residue. This is the first report detailing orlistat–lipase binding. It provides structure-based information on the production of potent anti-SAL drugs and lipase inhibitors. These results also indicated that orlistat can be repositioned to treat bacterial diseases.

## Introduction

*Staphylococcus aureus* (*SA*) can cause skin, throat, and digestive tract infections. *SA* is a native bacterium that resides in the nasal cavity, respiratory tract, and skin, and infection typically results in a localized collection of pus^1,2^. *SA* is related to several diseases and produces various types of pathogenic toxins^3^. Methicillin-resistant *SA* (MRSA) is one of the most well-known “super bugs,” which has developed resistance to almost all current antibiotics^4,5^. It is a common nosocomial infection that presents a serious risk to those with weak immune systems such as children and the elderly^6^. Identifying novel, effective drugs is of great importance for treating MRSA-related diseases.

**Figure 1:**
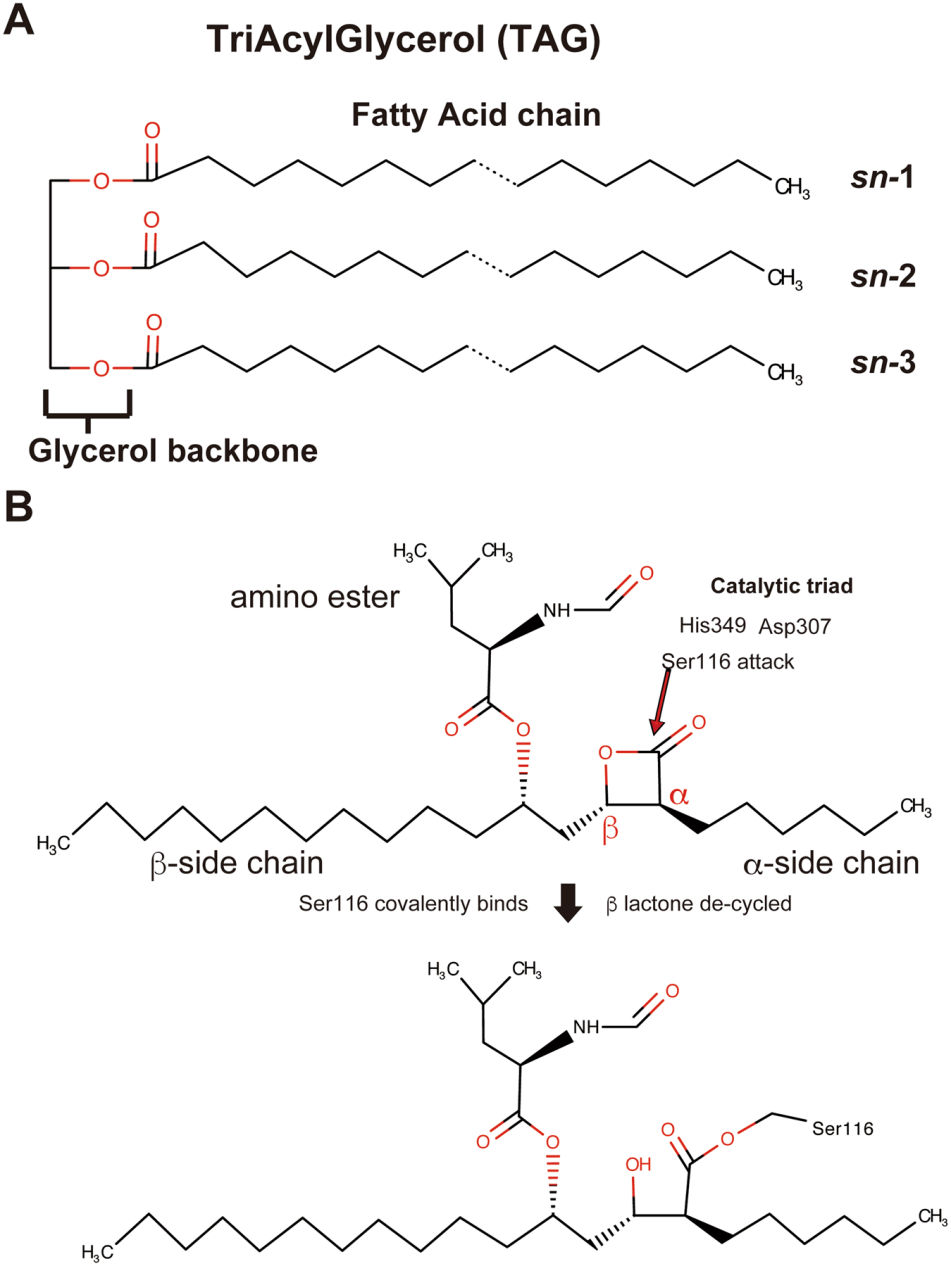
Chemical structures of triacylglycerol and orlistat. Triacylglycerol structure comprises one glycerol molecule and three fatty acids. (**B**) Proposed mechanism of orlistat binding. The catalytic triad His349, Asp307, and Ser116 attack orlistat. β-Lactone is hydrolyzed by Ser116 of SAL. The oxygen atom of Ser116 is covalently bound to the carbonyl carbon atom.

*SA* lipase (SAL, also known as glycerol ester hydrolase), a triacylglycerol esterase (Fig. 1A), shows strong cytopathic activity in host cells^7^. SAL is a potential target of anti-*SA* drugs in skin diseases^8^. SAL degrades lipids, which attack pathogenic bacteria^9,10^. By reducing the effect of immune-responsive lipids, *SA* colonization increases on the skin surface or inside the body. It has also been reported that some inhibitors such as farnesol decrease *SA* production^11^. Recently, Chen *et al*. observed that SAL released by *SA* inhibits the activation of innate immune cells and interferes with the host immune system to affect innate immune recognition in the microbe^12^.

SAL, an ester bond-hydrolyzing enzyme, is secreted in prepolypeptide form containing 690 amino acid residues and is processed into its mature form containing 394 amino acid residues. Its active site is composed of a typical catalytic triad including Ser116, Asp 307, and His349 residues, similar to other serine hydrolases.

Very few specific SAL-related bacterial lipase inhibitors have been identified to date^10,11^; tetracycline is a known inhibitor of *Staphylococcus epidermidis* lipase^13^. An example of a SAL inhibitor is farnesol, which is a competitive inhibitor^11^. However, this monoterpenoid weakly inhibits SAL (IC_50_ value, 0.57 mM). These inhibitors were not so potent against SAL-related bacterial lipases. We screened various chemical libraries and discovered that the anti-obesity drug orlistat is a potent SAL inhibitor with an IC_50_ value of 2.4 μM.

Orlistat belongs to the lipstatin family, derived from the gram-positive bacterium *Streptomyces toxytricini*^14^. The IUPAC name of orlistat is (2*S*)-1-[(2*S*,3*S*)-3-hexyl-4-oxooxetan-2-yl]tridecan-2-yl (2*S*)-4-methyl-2-(2-oxoethyl)pentanoate (Fig. 1B). Its Y-shaped chemical structure is divided into three fragments: amino ester moiety (N-formyl- L-leucine substituent extending off the C5 carbon atom), α-heptyl alkyl tail, and long β-tetradecanyl alkyl chain (Fig. 1B)^14,15^. Orlistat is a potent inhibitor of human gastric and pancreatic lipases that play an important role in dietary fat digestion. It is currently available in the market as an anti-obesity drug (Xenical or Alli), which acts locally to block gastric lipases that are crucial for the digestion of long-chain triglycerides^15^. However, there is no detailed structural information on how orlistat molecules bind to these lipases.

We discovered that orlistat is a potent SAL inhibitor at µM concentrations, and we succeeded in obtaining its crystal structure complex. Herein, we also reported 3D structures of native SAL, the inactive S116A mutant, and SAL–orlistat complex. Based on this binding of SAL with orlistat, we predicted the binding manner of orlistat with mammalian gastric lipase. These structures will help in the development of anti-SAL drugs.

## Results

### Structure determination of native SAL, inactive S116A mutant, and SAL–orlistat complex

**Figure 2:**
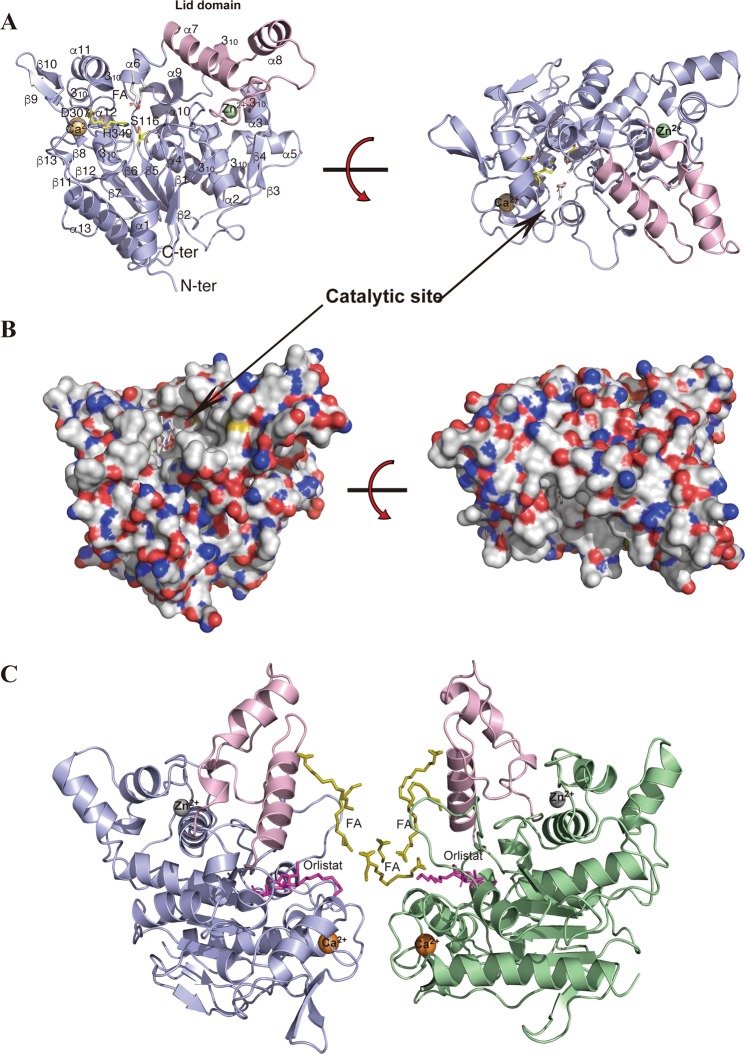
Crystal structure of native SAL and the SAL–orlistat complex. (**A**) Schematic of the SAL structure. α-Helices and β-strands are shown in blue; the lid domain is shown in pink. Fatty acid fragment molecules are shown as sticks. The calcium and zinc ions are shown in orange and green, respectively. (**B**) Molecular surface depiction of native SAL with positively charged areas shown in blue and negatively charged areas in red. (**C**) The dimeric form of the SAL complex with orlistat and fatty acids. Each monomer is shown in blue (chain A) and green (chain B), respectively. The lid domain is shown in pink. The orlistat and fatty acid fragment molecules are shown as sticks in magenta and yellow, respectively. All images of molecular structures were prepared using *PyMOL* (http://www.pymol.org/2).

This report describes the crystal structures of native SAL, inactive S116A mutant, and SAL–orlistat complex at resolutions of 2.08, 2.6, and 2.23 Å, respectively (Table 1). The crystal structure of native SAL was determined using MR techniques. The *Staphylococcus hyicus* lipase (SHL) chain A of 2hih^16^, which shares 47.7% identity with that of SAL, was used as a search model using the *CCP4 Phaser* program^17,18^. The MR solution automatically allowed unequivocal tracing of the two defined chains A and B (each chain with amino acid residues 4–385 was unambiguously determined with the exception of the His-tagged linker peptides N-terminus 1–3 and C-terminus 386–394, which are not visible) and was subsequently refined at a resolution of 2.08 Å using *REFMAC5*^19^. Five percent of the unique reflections were used to monitor the free *R*-factor. Final values of the general *R*-factor and free *R*-factor up to a resolution of 2.08 Å were 21.3% and 23.5%, respectively (Table 1). The dimeric refined model comprised 764 amino acids with 76 solvent molecules with 2 zinc and 2 calcium ions and 12 fatty acid fragments of various lengths (Fig. 2A–C).

**Table 1:** Data collection, processing, and refinement.

	Native SAL	Inactive S116A mutant	SAL–orlistat complex
PDB ID	6KSI	6KSL	6KSM
Diffraction source	BL44XU/SPring-8	BL44XU/SPring-8	BL44XU/SPring-8
Wavelength (Å)	0.90	0.90	0.90
Temperature (K)	100	100	100
Detector	Eiger X 16M	Eiger X 16M	Eiger X 16M
Crystal–detector distance (mm)	300	380	300
Rotation range per image (°)	0.1	0.1	0.1
Total rotation range (°)	180	180	180
Exposure time per image (s)	0.1	0.1	0.1
Space group	*P*4_1_22	*P*6_1_22	*P*4_1_22
a, b, c (Å)	129.9, 129.9, 251.2	164.2, 164.2, 233.1	132.5, 132.5, 248.2
α, β, γ (°)	90.0, 90.0, 90.0	90.0, 90.0, 120.0	90.0, 90.0, 90.0
Mosaicity (°)	0.044	0.063	0.049
Resolution range (Å)	50–2.08 (2.21–2.08)	50–2.59 (2.75–2.59)	50–2.23 (2.37–2.23)
Total No. of reflections	1761937	1155509	1467858
No. of unique reflections	246153	109044	205262
Completeness (%)	99.9 (99.3)	99.8 (98.7)	99.7 (98.5)
Redundancy	7.2 (7.1)	10.6 (10.4)	7.2 (6.6)
〈*I*/σ(*I*)〉	11.6 (0.7)	16.8 (0.9)	10.6 (0.7)
*R*_meas_	0.089 (2.566)	0.083 (2.138)	0.125 (2.573)
CC_1/2_	0.999 (0.481)	0.999 (0.523)	0.999 (0.424)
Refinement	Refmac	Refmac	Refmac
Resolution range (Å)	50-2.08	50-2.59	50-2.23
*R*-factor/free *R*-factor (%)	21.3/23.5	21.3/24.6	20.8/24.0
No. of atoms	6246	6110	6266
No. of solvent atoms	78	12	62
No. of ligands	16	9	13
Ramachandran distribution (% favored, allowed, outlier)	98.0, 2.0, 0	95.0, 5.0, 0	96.8, 3.2, 0
RMS bonds (Å), angles (°)	0.028, 2.91	0.020, 2.36	0.026, 2.69
Average B value (Å^2^)	47.1	65.0	45.8

Values for the outer shell are given in parentheses. Completeness for all reflections and for the highest-resolution shell in parentheses.

Stereochemistry checks indicated that the refined models were in good agreement with the expectations with these models within this resolution range (Table 1). The asymmetric unit contained two SAL molecules (Fig. 2C).

Structures of Inactive S116A mutant and SAL–orlistat complex were also determined with the MR technique using the *CCP4 Phaser* program^18^. The SAL–orlistat complex, which was isomorphous with native SAL, showed strong positive electron density maps of orlistat molecules near the active sites of chains A and B. The crystallization condition of the S116A mutant crystal was different than the native SAL crystal, with the former displaying a hexagonal system and with two molecules in the asymmetric unit.

### Overall structure

**Figure 3:**
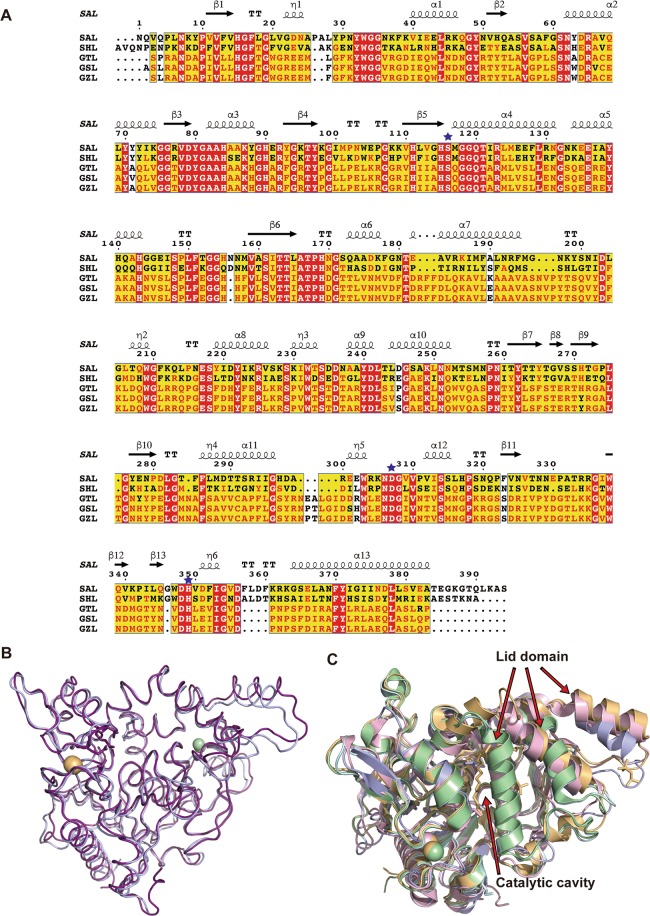
(**A**) Sequence alignments of SAL, SHL, GTL, GSL, and GZL. Multiple sequence alignment of SAL and these structural homologous lipases was performed with *ClustalW*, followed by the *ESPRIT* program. The secondary structure elements of SAL are given above their corresponding sequences. Arrows and springs above the sequences indicate β-strand and helical conformations, respectively. The catalytic triad residues are marked with an asterisk. (**B**) Superimposition of SAL and SHL structures are shown in blue and pink, respectively. (**C**) Structural comparison of SAL with other homologous lipases. SAL, SHL, and GTL are shown in their open-lid form in blue, pink, and orange, respectively. GSL and GZL are shown in the closed form in green and cyan, respectively. Fatty acid fragment molecules are shown as sticks. The lids of GSL and GZL overlap with the position where fatty acids are localized in SAL.

The SAL crystal structure exhibited a typical α/β-hydrolase fold comprising a central twisted β-sheet flanked by α-helices on both sides. The refined model comprised 13 β-strands, 13 α-helices, and 6 3_10_ helices (Figs. 2A and 3A). The SAL crystal structure contained a conserved catalytic triad (Ser116, His349, and Asp307) (Fig. 3A), which was located at the concaved cleft (Fig. 2A). SAL had a unique shape like a cow face with two metal eyes and two ears formed by β-strands and α-helices with a deep central cavity as the catalytic site (Fig. 2A,B). The cavity within the substrate-binding domain contained two large hydrophobic alkyl chain-binding pockets and a shallow, more polar third pocket with a Ser116 catalytic center capable of binding a fatty acid or lactone ester (Fig. 2B).

### Catalytic triad

Ser116, a catalytic residue, is important for cleaving substrates and has the same catalytic mechanism as other serine hydrolases. The nucleophile Ser116 within the consensus GXSXG lipase motif was located at the “β-elbow” loop between β-strand 5 and α-helix 4 of the α/β hydrolase core^20^. The backbone nitrogen atoms Met117 and Phe17 formed an oxyanion hole that can stabilize the intermediate transition state during catalytic reactions. Ser116 was located at the bottom of an approximately 11-Å-deep and 23-Å-wide cavity. The entrance of this cavity was formed by hydrophobic clusters (Phe17, Leu18, Ty29, Pro30, Phe178, Met188, Leu287, Phe286, Val350, and Val355).

### Metal-binding site

The zinc- and calcium-biding sites of lipases are highly conserved across many types of species^16^. The amino acid residues that recognize these metals are also highly conserved in various types of lipases. These two metal ions were located near the active site pocket opposite each other, possibly to stabilize the 3D SAL structure.

The SAL zinc ion was primarily surrounded by two histidine (His84 and His90) and two aspartate (Asp64 and Asp236) residues, showing same binding as SHL (Supplementary Fig. 1A). These four residues are highly conserved among various homologous lipases^21^. The two tyrosine residues (Tyr80 and Tyr88) surrounded the zinc ion with benzene rings (Supplementary Fig. 1A).

The SAL calcium ion was primarily surrounded by four aspartate residues (Asp348, Asp351, Asp356, and Asp359), similar to SHL (Supplementary Fig. 1B)^16^. The Gly283 carbonyl oxygen was also present at a resolution of 2.2 Å, which has the same form as SHL and other lipases^16^.

### Dimerization complex

Similar to SHL, SAL was observed in dimeric form in the crystal structure, but the combination of dimer molecules was different than that in SHL^16^. In the native SAL crystal structure, dimer interactions were typically hydrophobic (Fig. 2C). There were 12 interacting residues, located near each calcium-binding site. Calcium ions were located at a distance of 16.5 Å from each dimer. Hydrophobic clusters were constructed at the loop regions surrounding calcium ions by Asp281, Leu282, Phe286, Phe357, Leu358, and Phe360 (Fig. 2C). The S116A mutant crystal had a different crystallization condition than the native crystal, as shown by the distinct dimeric form.

Remarkably, fatty acid branches were located between the dimer interface, which should stabilize the dimeric form of SAL. Fatty acids may have bound during the production of SAL in *Escherichia coli* cultivation and continued to bind to SAL even after the three-step chromatography. Crystal packing was stabilized by the insertion of fatty acids between molecules. Gel filtration showed that the dimeric form was stable in solution form at pH 7.5 in a physiological buffer (Supplementary Fig. 2).

### Lid domain

Similar to other lipases, SAL had a “lid domain” to regulate the catalytic cavity between the open and closed forms^22^. The lid domain was formed from Thr181 to Thr233, and it was mainly constructed in a loop form by two α helices, i.e., helix 7 and helix 8, which were located on the surface of the SAL structure (Fig. 2A,C). The native SAL structure was observed as an open form (Fig. 2A). The SAL–orlistat complex also had an open form, with the lid region located as far as possible from the catalytic cavity (Fig. 2C). The overall structures of SAL and SHL were relatively similar, except the lid domain, which was due to the differences in crystal packing between SAL and SHL (Fig. 3B).

Figure 3C shows the overlay of various lipases in the open and closed forms^23–25^. In lipases with closed forms, the two lid helices shifted toward the catalytic cavity, and the catalytic cavity was completely occupied by long α-helix 7 (Fig. 3A,C)^22^. Remarkably, the lid of the opened form, the α-helix 8 of SAL, was approximately 13 Å outward from the closed form position, thereby allowing substrates or inhibitors to enter the catalytic cavity (Fig. 3C).

### Characterization of SAL inhibition by orlistat

**Figure 4:**
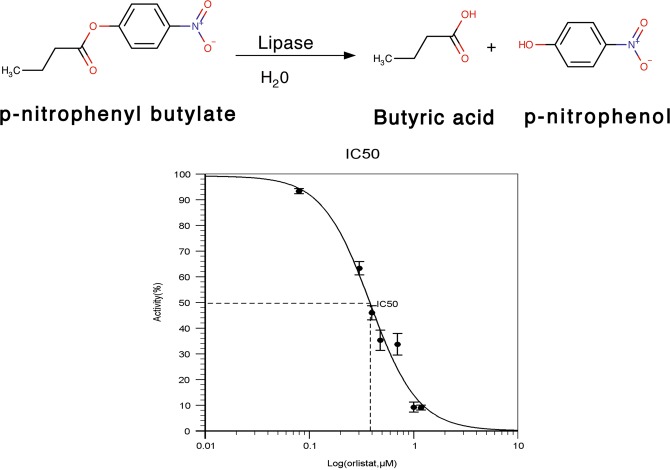
Inhibition of SAL by orlistat. SAL activity toward *p*-nitro phenylbutyrate. Enzymatic activity was measured by monitoring *p*-nitrophenol production. Relative activities were measured at various inhibitor concentrations. Data are shown as mean ± s.d. (n = 3). IC_50_ value was determined using the IC_50_ calculator (https://www.aatbio.com/tools/ic50-calculator).

The potent inhibitors of SAL were searched via chemical libraries. We also tested commercially available lipase inhibitors. *p*-Nitrophenyl butyrate ester was used in an *in vitro* lipase assay, and the absorbance of liberated *p*-nitrophenol was monitored continuously at a wavelength of 405 nm. The assay revealed that lipase activity was inhibited in an orlistat dose-dependent manner, and the IC_50_ value was calculated as 2.4 µM (Fig. 4).

### Inhibitor–fatty acid binding

**Figure 5:**
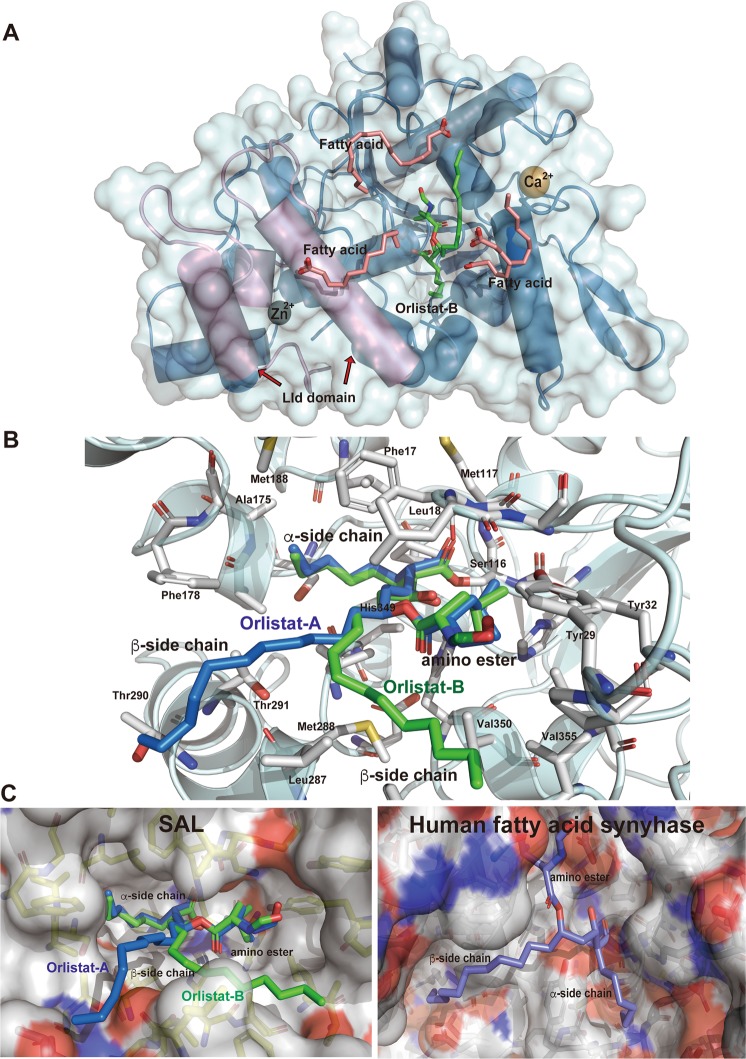
Binding interaction between SAL and orlistat. (**A**) Overall structure of SAL complex with orlistat and fatty acids. (**B**) Close-up view. The recognition mode of orlistat at the SAL catalytic site. Two orlistat molecules are overlapped in blue (chain A) and green (chain B) in the stick model. The residues, which indicate orlistat recognitions, are presented as stick models in white. (**C**) Orlistat-binding regions are highlighted as surface models. (left) Orlistat (blue and green sticks) and SAL complex; (right) Orlistat (blue stick) and the thioesterase domain of human fatty acid synthase complex. Positively charged areas are shown in blue, and negatively charged areas are shown in red.

The crystal structure of the SAL–orlistat complex showed an inhibitor-binding site and atomic-level details of binding interactions (Fig. 5A,B). Notably, the β-lactone rings of orlistat molecules were decyclized by their hydrolyzing ring cleavage reaction with SAL on chains A and B. Ser116 attached the carbonyl carbon atom of the orlistat tetracyclic rings at the active sites (Figs. 1 and 5B). Then, orlistat covalently bound to Ser116, with the oxygen atom of Ser116 and orlistat maintaining the bond. The same binding process has been shown for orlistat and the thioesterase domain of the human fatty acid synthase complex, wherein the β-lactone ring of orlistat was hydrolyzed and the carbonyl oxygen covalently bound to catalytic serine residues^26^. However, the mode of interaction of SAL with orlistat is different from that previously determined for human fatty acid synthase complexes (Fig. 5C).

**Figure 6:**
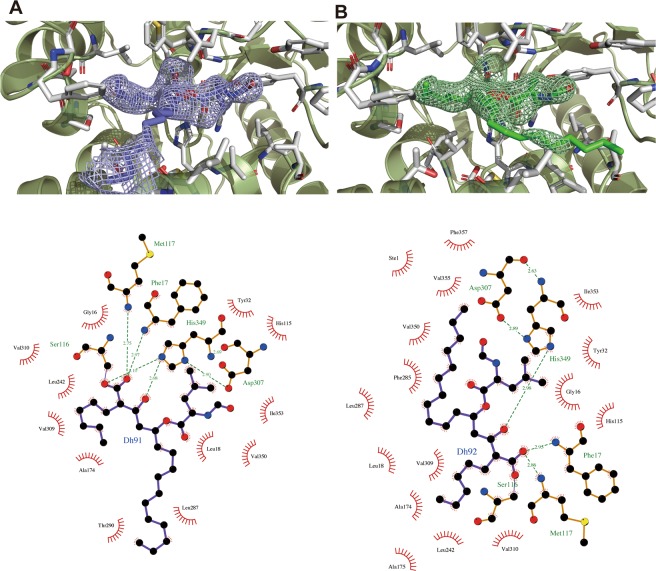
Omit maps of orlistat are shown for chains A and B. (**A**,**B**) The residues that indicate orlistat recognitions are presented as stick models in white. The key interactions of orlistat at the SAL active site are shown using a *LIGPLOT* + diagram^39^.

SAL monomer bound one orlistat molecule each in a similar manner. Both β-lactone rings were decyclized, but minor differences were observed in the long alkyl β-side-chain moieties (Figs. 5B, 6A,B). Seventeen data sets of the SAL–orlistat complex crystal were collected. All electron density maps for the alkyl β-side chain were not strong, indicating the flexibility of this portion. Two possible conformations of this moiety were observed (Fig. 6A,B). The Ser116-binding decyclized region and amino ester portions were tightly bound to the SAL active site, with a clear electron density map (Fig. 6A,B). The binding mode of orlistat to SAL active sites was divided into three main parts, which matched orlistat’s unique Y-shaped form (Fig. 1). Orlistat has a long β-chain and short α-chain, which are constructed by alkyl hydrophobic carbon atoms and are fitted to the hydrophobic grooves of the SAL structure (Fig. 5C). The decycled β-lactone presented as one hydroxy group and one carboxylate moiety with extended forms (Fig. 1B). The oxygen atom of the hydroxy group is hydrogen bound to the His349 side-chain nitrogen atom (2.67 Å) (Figs. 5B and 6). The Ser116 side-chain residue covalently bound to the carboxylate group and shared one oxygen atom with both SAL and orlistat (Figs. 1, 5B, and 6). The other oxygen of the decycled carboxylate group formed hydrogen bonds with two nitrogen atoms of the main chain Phe17 (2.97 Å) and Met117 (2.75 Å), which form the oxyanion hole (Fig. 6A).

The amino ester moiety of orlistat was a modification of the leucine compound (Fig. 1B) and was located close to the enzyme in a configuration typically seen during hydrophobic contact. The leucyl moiety made hydrophobic contact with Tyr32, Il353, His115, and Val350. The terminal N-formyl carbonyl group of the amino ester moiety was located in the hydrophobic pocket formed by Leu18, Tyr29, Pro30, Tyr32, Val350, Ile353, and Val355 (Fig. 5B).

The α-side chain of orlistat was also primarily bound to a hydrophobic pocket, which was formed by Ala174, Ala176, Pro168, Leu242, Val309, Val310, and partially Phe17 at the bottom of its deep active site (Fig. 5B). Two possible conformations were observed on the β-side chain of orlistat. One was extended (in chain A), whereas the other was bent at an angle of 90° (in chain B) (Fig. 6A,B).

The β-chain of orlistat on chain A contained the hydrophobic residues Leu18, Phe178, Leu287, Thr290, Thr291, and Val309. Its terminal alkyl chain was outside the active site (Fig. 6A). The β-alkyl chain of orlistat on chain B, which was parallel to the leucyl moiety, demonstrated hydrophobic contact with Leu18, Phe285, Leu287, Val350, Val355, and Phe357 (Fig. 6B). The fatty acids of the native SAL crystal also bound at the same site. These formed two alkyl chain recognition sites and a leucyl moiety site (Fig. 6).

**Figure 7:**
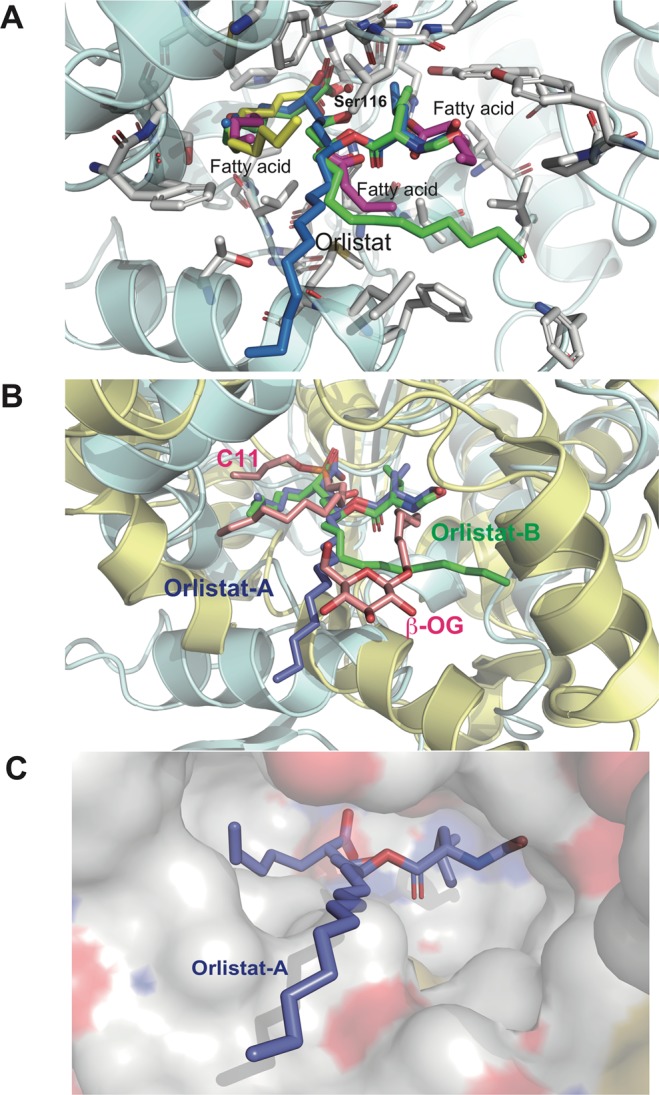
Superimposition of SAL-binding molecules. The orlistat complex (blue and green) and fatty acids fragments in native (magenta) SAL and S116A mutant (yellow). The key residue side chains are shown as sticks, and the protein backbone is shown schematically in light cyan. The catalytic triad residues are shown as sticks (separated structures shown in Supplementary Figure 3). (**B**) Superimposition of the orlistat complex of SAL (blue) and the phosphonate inhibitor C11 and β-octyl glycoside complex of DGL. SAL (light blue) and DGL (light yellow) overlap. (**C**) The predicted model of orlistat and DGL (surface model) binding mode. Orlistat molecules are shown as sticks in blue. Positively charged areas are shown in blue, and negatively charged areas are shown in red.

An inactive Ser116 mutant with Ala substitution, which lost PNPB-hydrolyzing activity, was also constructed. The S116A mutant crystal structure also bound to fatty acids near the Ser116 residue (Fig. 7A, Supplementary Fig. 3B). Wild-type S116A and S116A mutant differentially bound to fatty acids at the active site due to amino acid differences between Ser and Ala. The carboxylate moieties of fatty acids shifted closer to Ala116 in the mutant than in the wild type (Fig. 7A).

Fatty acids were observed in the native SAL, S116A mutant, and SAL–orlistat complex structures (Fig. 7A, Supplementary Fig. 3). In the native SAL crystal, three fatty acids were observed near the active site, whereas only one fragment was bound to S116A mutant. SAL specifically hydrolyzes triacylglycerol (Fig. 1A), and these three fatty acids may reflect a triacylglycerol position for hydrolysis (Fig. 7A, Supplementary Fig. 3).

In some cases, when using PEG solution for crystallization, multiple rounds of analysis were required to detect PEG fragments in structures. The native and S116A mutant crystals did not use PEG under the crystallization condition or during cultivation or purification. Therefore, the observation that SAL binds to fatty acids is interesting because they may have been derived from culture broths of *E. coli*. Fatty acids were present at the same position on the long β-side and short α-side chains. The fatty acid bound in the S116A mutant was located on the short α-side chain and was almost 12 carbon atoms in length (Fig. 7A, Supplementary Fig. 3). These results strongly supported that SAL has a high affinity for fatty acids and hydrolyzes them to reduce the effect of immune-responsive lipids^9,12^.

## Discussion

*DALI* showed that several bacterial lipase structures match the 3D SAL structure well^27^, with the best-matched lipase being SHL (PDB ID: 2hih; RMSD, 1.3 Å), followed by *Geobacillus thermocatenulatus* lipase (GTL; PDB ID: 5ce5; RMSD, 1.8 Å), *G*. *stearothermophilus* lipase (GSL; PDB ID: 1ji3; RMSD of 2.3 Å), and *G*. *zalihae* lipase (GZL; PDB ID: 3umj; RMSD 2.3 Å)^16,23–25^. Multiple sequence alignment of SAL and structural homologous lipases was performed using *ClustalW*, and Fig. 3A was constructed using the *ESPIRIT* program^28^. The 3D structures of these lipases were also determined. They had no similarity in terms of amino acid sequences with human gastric lipase (HGL; data not shown).

In these alignments, SAL, SHL, and GTL crystal structures had an open-lid conformation (Fig. 3C). Among these, GSL and GZL crystal structures had a closed conformation^24,25^. It has been observed that structures with open-lid forms may bind ligands in the lid domain, possibly inhibiting lid closure. Although fatty acids bound to SAL, no small molecules were observed bound to SHL due to low-resolution analysis (approximately 2.8 Å). PEG and MPD molecules bound to GTL. Further, open forms of lipases have PEG or fatty acid molecules inserted within the active site cleft. These molecules prevented lid movement at α-helix 7, resulting in an open-lid conformation in crystal structures (Fig. 3C).

The native SAL crystal structure has the same fold as the SHL crystal structure, which has phospholipase activity^16^. This shape may be suitable for accessing membrane phospholipids. When SAL attaches to the membrane, it can hydrolyze the proximal lipid chains of the membrane.

Various fatty acids bind to the active and nonactive sites of the SAL crystal structure in its native form (12 fatty acids) and the SAL–orlistat complex (7 fatty acids). The other four long fatty acids were also found bound far from active sites on chains A and B of the SAL structure, particularly at the lid of α-helix 7 and helix 8. This also blocked the lid domain from moving forward to the active site, thereby maintaining the lid in its open form. These surplus strong findings of electron density maps of fatty acids were also observed for the SAL–orlistat complex structure covering the α-helix 7 lid, which seemed to be preventing its movement.

SAL shares 54% identity and 67% similarity with the SHL primary structure, respectively. These two lipases possessed the same whole structures and have almost identical secondary folds. The difference lies in helix 8, which is located at the lid domain (Fig. 3B) near the zinc-binding site, and is shifted by approximately 6 Å between each other. The lid domain is not close to the catalytic pocket in the SAL structure, whereas the SHL structure is externally opened with α-helix 10. It was observed that the structural differences in these loop regions occurred due to crystal packing. The packing combinations of SAL and SHL were also different from each other.

This study succeeded in determining the 3D structure of the SAL–orlistat complex. However, it remains unclear how orlistat binds to human lipases based on the current SAL–orlistat complex. No similarity was found with HGL in terms of amino acid sequence or 3D structure. The 3D alignment of SAL to HGL (PDB ID: 1hlg) showed that the small α-helix of HGL overlapped to interfere with the SAL active sites and disturbed the binding mode of orlistat, which led HGL to have a closed form^29^.

Although HGL has been reported as a closed form, the dog gastric lipase (DGL; PBD ID: 1k8q) complex has been reported as an open form in the presence of the phosphonate inhibitor C11^30^. The current study compared the DGL–C11 complex with the SAL–orlistat complex. Using the *SSM* module of the Coot program^31^, the DGL complex structure was fitted to the SAL–orlistat complex structure (Fig. 7B). The overlapped orlistat molecule showed a similar fit between the active site of DGL and the SAL-binding mode (Fig. 7B). In the DGL– C11 complex, β-octylglucoside also bound to the active site^30^ and was well-aligned with the β-chain moiety of the orlistat-binding site (Fig. 7B). C11 covalently bound to catalytic Ser153 of DGL, which also overlapped with the α-chain moiety in orlistat. The docking study also showed that the binding interaction between orlistat and DGL is similar to that between orlistat and SAL (Fig. 7C). Therefore, the interactions between orlistat and HGL are possibly similar to that between orlistat and DGL.

Although it has not yet been determined how orlistat binds to human lipases, this study showed how orlistat bound to a bacterial lipase derived from *SA*, which causes human skin diseases and MRSA proliferation. These results suggest that orlistat is repositioned in dermatosis or MRSA-related diseases.

In summary, we found that orlistat is a potent SAL inhibitor, provided a novel 3D structure of SAL, and characterized the detailed molecular interactions between orlistat and SAL. Biochemical analyses and crystallographic studies of SAL and its inactive mutant allowed for the understanding of the catalytic mechanism of SAL. Using this structural information of SAL, highly potent structure-based drugs can be designed and developed for the treatment of *SA* skin disease and MRSA-related disease.

## Materials and methods

### Cloning and expression of SAL

The SAL gene was cloned into a pCold II vector (Takara Bio) and amplified by PCR with the KOD FX DNA polymerase (Toyobo) using chromosomal DNA as previously reported^32^. The plasmid for the expression of the Ser116Ala mutant was constructed using site-directed mutagenesis with 5′-CTTGTAGGGCATgcTATGGGTGG-3′ as the mutagenesis primer. The PCR reaction mixture included 0.25 U Tks Gflex DNA polymerase (Takara Bio), 0.2 mM deoxynucleotide triphosphate, polymerase buffer, 0.2 µM primer, and 20 ng *pCold II-mSAL* plasmid DNA solution as the template DNA in a total volume of 50 µL. The samples were preheated at 94 °C for 30 s, followed by 25 cycles of amplification under the following conditions: denaturation at 94 °C for 30 s, annealing at 55 °C for 1 min, and elongation at 72 °C for 6 min using a thermal cycler (GeneAtlas). After PCR, the products were digested by 10 U *Dpn*I at 37 °C for 1 h. The *Dpn*I-treated PCR products were introduced to *E. coli* DH5α cells using chemical transformation. The consequent plasmid was extracted from the transformant, and the mutation in the plasmid was confirmed using DNA sequencing. Finally, the constructed plasmid, termed *pCold II–S116A mSAL*, was introduced into the *E. coli* BL21(DE3) strain (Novagen).

### Protein expression and purification

Recombinant SAL and S116A-SAL were expressed in *E. coli* and purified as described previously^32^ with three-step chromatography. The eluted protein containing SAL was further purified using size-exclusion chromatography to analyze its molecular size in solution (Supplementary Fig. 2). The purified SAL (10 mg/mL) was injected at 20 °C into the GE Superdex 200 increase 10/300 GL column. Chromatography was performed with 50 mM Tris-HCl (pH 7.5) containing 0.2 M NaCl at a flow rate of 0.5 ml/min, using BSA as a standard protein for calibration. The protein was concentrated to 30 mg mL^−1^ using the VIVASPIN 15 turbo concentrator (Sartorius, Germany) and sterilized by filtration through a 0.1-µm Ultrafree-MC device (Merck Millipore, Germany). The homogeneity of the purified preparation was confirmed by 12% SDS-PAGE.

### Determination of specific activity

Enzymatic activities were determined using *p*-nitrophenylbutyrate (pNPB) ester substrate as previously reported^32,33^. Enzyme concentration was adjusted to 0.002 mM and pNPB concentration to 0.08 mM. Each assay was performed at 25 °C with 50 mM HEPES (pH 7.5) containing 10% dimethylformamide in 1-ml cuvettes. Various concentrations of orlistat (0–0.01 mM) were prepared to determine IC_50_ values. Before starting the SAL/pNPB reaction, SAL was incubated with orlistat for 15 min. Reaction curves were obtained following the addition of substrate at 25 °C. The absorbance of liberated *p*-nitrophenol was monitored continuously at a wavelength of 405 nm, and the initial linear velocity was used to calculate the specific activity of SAL. An experimentally determined absorption coefficient of 18.5 mM^−1^ cm^−1^ was used for all calculations^33^. Determination of the initial rate was performed at least thrice. IC_50_ values were determined with data averaged at each concentration using an IC_50_ calculator (https://www.aatbio.com/tools/ic50-calculator). The various orlistat concentrations inhibited SAL activities by more or less than 50% (Fig. 4).

### Cocrystallization

For crystallization experiments, a 30 mg mL^−1^ solution of the recombinant SAL was prepared in 0.2 M NaCl and 10 mM Tris-HCl at pH 8.0. The inhibitor concentration was adjusted to 5–10-fold molar equivalents. Concentrated SAL was incubated overnight with 5–10-fold molar excess of the inhibitor dissolved in DMSO.

Crystallization trials were set up as sitting-drop vapor-diffusion experiments at 295 K on Cryschem crystallization plates. Initial screening was performed using the sparse matrix method^34^ with commercial crystal screening kits (Hampton Research, Wizard). Several trials were performed at varying protein concentrations, precipitant concentrations, and pH values to optimize experimental conditions.

### Data collection and processing

X-ray diffraction data of SAL crystals were collected at 100 K in a flowing nitrogen stream. The mother liquor solution was supplemented with 30% glycerol as a cryoprotectant. X-ray data sets were collected from a single crystal using the beamline BL41XU and BL44XU at SPring-8. The X-ray wavelength was tuned to 0.9 Å. Further, the oscillation angle was 0.2°, and the crystal-to-detector distance was approximately 300 mm. Diffraction images were integrated and scaled using the *XDS* program package^35^ with the *KAMO* automatic program at SPring-8^36^. The resolution limits were defined as CC_1/2_ of approximately 0.5^37^.

### Structure determination, model building, and refinement

Crystal structures were determined by molecular replacement using *Phaser*^20^. Model building and inspection were based on *Coot*^31^ and refined by *Refmac5* on *CCP4*^19^. All images of molecular structures were prepared using *PyMOL* (http://www.pymol.org/2).

### Molecular Docking of orlistat in DGL

Structure refinement and energy minimization of the DGL–orlistat complex (Fig. 7B) was conducted using the *YASARA* Energy Minimization Server^38^.

### Accession codes

The atomic coordinates and structure factors for native SAL, inactive S116A mutant, and SAL–orlistat complex have been deposited in the Protein Data Bank with accession codes 6KSI, 6KSL, and 6KSM, respectively.

## Supplementary information

Supplementary Information.
