# Supplementary material for: Crystal structure of pathogenic Staphylococcus aureus lipase complex with the anti-obesity drug orlistat
Source: Sci Rep. 2020 Mar 25;10:5469. doi: 10.1038/s41598-020-62427-8 (PMC7096528; doi:10.1038/s41598-020-62427-8)

Crystal structure of pathogenic *Staphylococcus aureus* lipase complex with the antiobesity drug orlistat

Kengo Kitadokoro[a]*, Mutsumi Tanaka[a], Takaaki Hikima[b], Yukiko Okuno[c], Masaki Yamamoto[b], Shigeki Kamitani[d]

[a] Faculty of Molecular Chemistry and Engineering, Graduate School of Science and Technology, Kyoto Institute of Technology, Hashigami-cho, Matsugasaki, Sakyo-ku, Kyoto 606-8585, Japan; kengo@kit.ac.jp

SUPPLEMENTARY RESULTS

Supplementary Figure 1. Omit electron density map around zinc and calcium ions.
A difference Fourier map (Fo − Fc) was calculated with zinc (A) and calcium ions (B) in a model of bound structure and shown as a blue mesh contoured at 5.0 sigma. Residues involved at the zinc and calcium-binding sites are labeled and shown as sticks, and the calcium ion is shown as a green sphere.

Supplementary Figure 2. Size-exclusion chromatogram of SAL after three-step purification. The single peak shows the dimeric form of SAL.

Supplementary Figure 3. Active site structures of three SAL structures. (A) Native structure complex with fatty acids (magenta). (B) S116A mutant structure complex with fatty acids (yellow). (C) Native structure complex with orlistat (blue). The catalytic triad residues His349, Asp307, and Ser116 (Ala116) are shown in blue sticks.

Supplementary material and methods

Inhibition studies of orlistat

SAL was inhibited by excess molar orlistat (final 2 mM); then, unbound orlistat was removed using a PD-10 column (GE Healthcare) and equilibrated with 10 mM Tris buffer (pH 8.0) supplemented with 0.2 M NaCl.

SAL protein solutions were assessed with SDS-PAGE (**Supplementary Figure 4a**) before and after PD-10 column elution. Then, the residual activities of SAL were detected by pNPB hydrolysis. Sample A is before elution and sample B is after elution using PD-10 column (**Supplementary Figure 4b**).

SI Figure 1

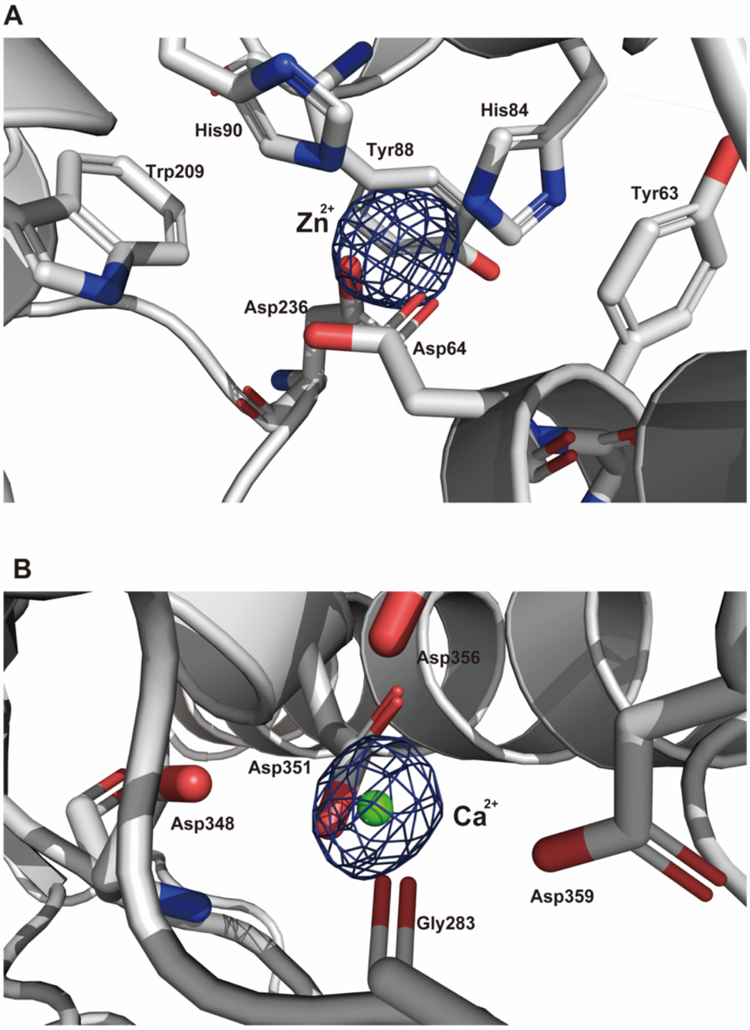

SI Figure 2

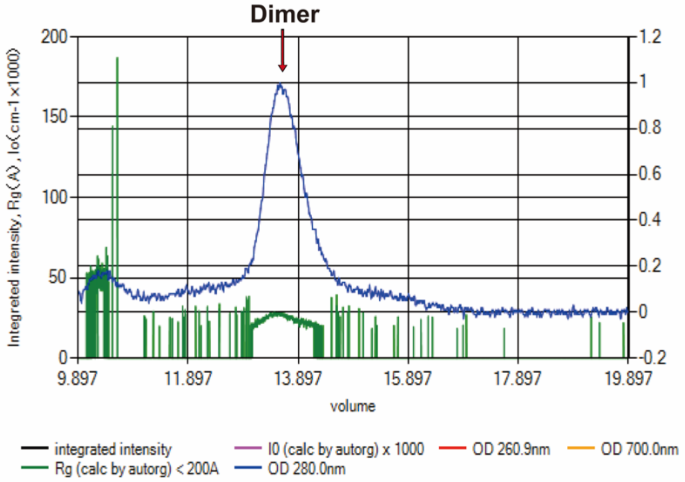

SI Figure 3

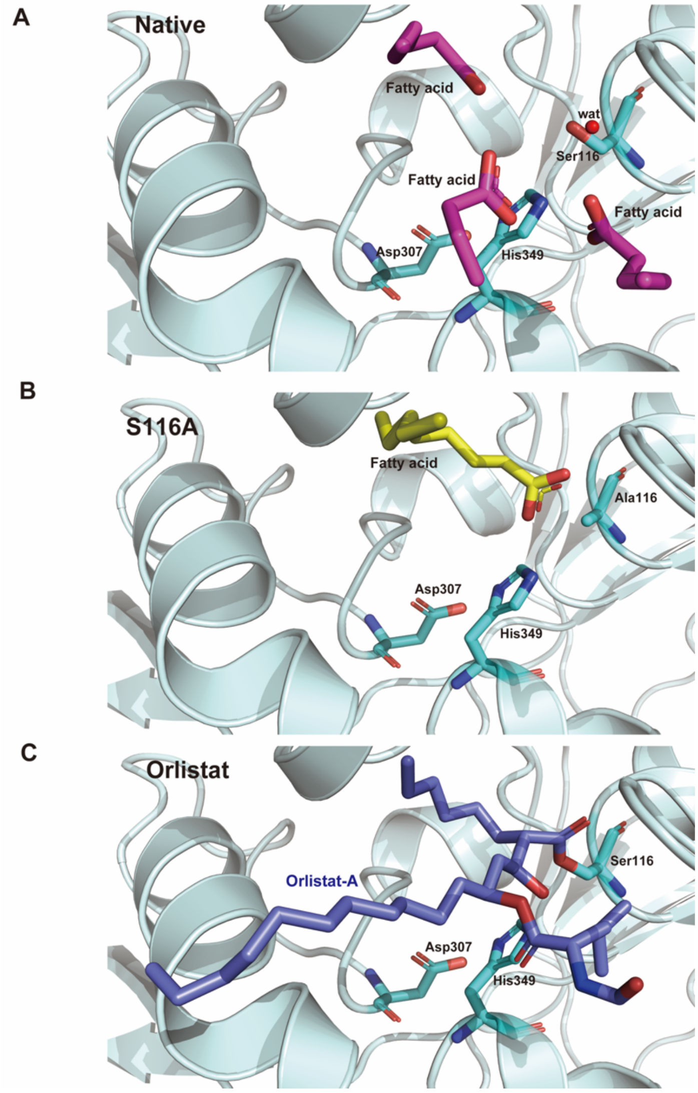

S1 Figure 4 (a) (b)

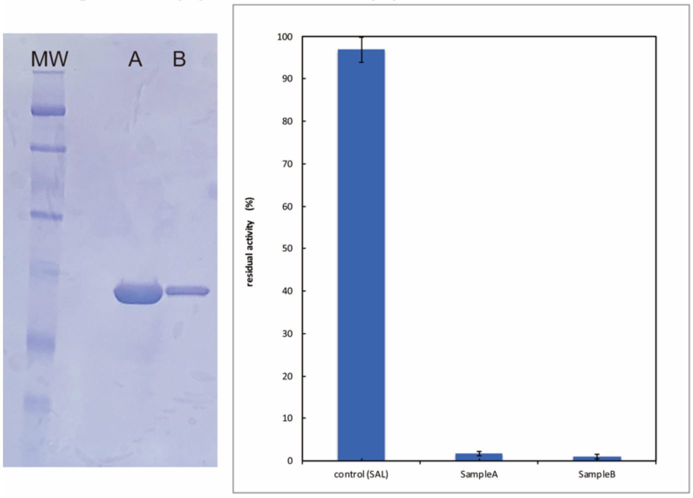

Supplement: Supplementary file 1 — Supplementary Information. [file 41598_2020_62427_MOESM1_ESM.pdf]
